# Author Correction: Biometrics’ new identity—measuring more physical and biological traits

**DOI:** 10.1038/s44319-025-00506-5

**Published:** 2025-06-16

**Authors:** Andrea Rinaldi

**Affiliations:** Freelance Science Writer, Cagliari, Italy

## Abstract

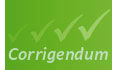

**Correction to:** *EMBO Reports* (2015) 17:22–26. 10.15252/embr.201541677 | Published online 14 December 2015

Figure 2 has been removed from the manuscript due to copyright reasons.

